# Correction: Development and *in vitro* and *ex vivo* characterization of a twin nanoparticulate system to enhance ocular absorption and prolong retention of dexamethasone in the eye: from lab to pilot scale optimization

**DOI:** 10.1039/d5na90032h

**Published:** 2025-05-02

**Authors:** Muhammad Sarfraz, Gautam Behl, Sweta Rani, Niall O'Reilly, Peter McLoughlin, Orla O'Donovan, Alison L. Reynolds, John Lynch, Laurence Fitzhenry

**Affiliations:** a Ocular Therapeutics Research Group (OTRG), Pharmaceutical & Molecular Biotechnology Research Centre (PMBRC), South East Technological University Waterford X91 KOEK Waterford Ireland muhammad.sarfraz@setu.ie; b Faculty of Pharmacy, The University of Lahore Lahore 56400 Pakistan; c UCD School of Veterinary Medicine Dublin Ireland; d UCD Conway Institute of Biomolecular and Biomedical Research, University College Dublin (UCD) Dublin D04 V1W8 Ireland

## Abstract

Correction for ‘Development and *in vitro* and *ex vivo* characterization of a twin nanoparticulate system to enhance ocular absorption and prolong retention of dexamethasone in the eye: from lab to pilot scale optimization’ by Muhammad Sarfraz *et al.*, *Nanoscale Adv.*, 2025, https://doi.org/10.1039/d4na01086h.

The authors regret that the incorrect spelling of the name of author Gautam Behl was used in this manuscript. The corrected spelling for this author name is as above. All affiliations for this author remain the same.

The Royal Society of Chemistry apologises for these errors and any consequent inconvenience to authors and readers.

